# Supplementary material for: Content-rich biological network constructed by mining PubMed abstracts
Source: BMC Bioinformatics. 2004 Oct 8;5:147. doi: 10.1186/1471-2105-5-147 (PMC528731; doi:10.1186/1471-2105-5-147)
Supplement: Additional File 2 — The original results of the above study (non-essential files are deleted to keep the file size under the limit set by BMC bioinformatics). [file 1471-2105-5-147-S2.bz2 › chilibotAdditionalFile2/dip30/711ID8679677E18048/html/PABA_PABB.html]

 


 **PABA** and **PABB** 
  
Found 23 abstracts in PubMed, retrieved 23.  
 

 What does Google say? 
 PDF only 
| .edu only 

---

**Interactive relationship** (e.g. stimulation, inhibition, etc)

**Neutral relationship**- We conclude that both  **PabA**  and  **PabB**  have cysteine residues which are essential for catalytic function and or for subunit interaction.  Ref: 7592344 J Bacteriol, 1995
- **PabA**  requires stoichiometric amounts of  **PabB**  to display glutaminase activity.  Ref: 8096767 Biochemistry, 1993
- **PabB**  forms a 1 1 complex with  **PabA**  to yield a glutaminase k cat of 17 min 1.  Ref: 1637823 Biochemistry, 1992
- A gene  **pabB**  encoding the aminase activity of p aminobenzoate  **PABA**  synthase in Lactococcus lactis subsp.  Ref: 8409921 J Gen Microbiol, 1993
- The cloned heterologous  **pabB**  DNA was expressed in amounts that allowed accumulation of excreted  **PABA**  in cultures of S. lividans transformants.  Ref: 8409921 J Gen Microbiol, 1993
- Similarly, while isolated  **PabA**  shows no tendency to form a glutamyl enzyme intermediate, the  **PabA**   **PabB**  complex forms a covalent intermediate with 14C glutamine on  **PabA**  that accumulates to 0.56 mol mol in hydrolytic turnover.  Ref: 1637823 Biochemistry, 1992
- **PabA**  is thus a conditional glutaminase, activated by 1 1 complexation with  **PabB** .  Ref: 1637823 Biochemistry, 1992
- Aminodeoxychorismate ADC synthase  [ **PABB** ]   [ **PABA** ]  converts chorismate and glutamine to ADC and glutamate and is composed of two subunits,  **PabA**  and  **PabB** .  Ref: 7592344 J Bacteriol, 1995
- Using purified  **PabB** , we have confirmed the recent suggestion that  **PabB**  needs an additional protein, enzyme X, to convert chorismate and NH3 to p aminobenzoate  **PABA** .  Ref: 2251281 Proc Natl Acad Sci U S A, 1990
- 4 Amino 4 deoxychorismate ADC synthase  [ **PABB** ]   [ **PABA** ]  converts chorismate and glutamine to ADC and glutamate, and is composed of two subunits,  **PabA**  and  **PabB** .  Ref: 8679677 Biochim Biophys Acta, 1996
- We report that  **PabA**  requires a preincubation with dithiothreitol for maximal activity as measured by its ability to support the glutamine dependent amination of chorismate by  **PabB** .  Ref: 7592344 J Bacteriol, 1995
- A comparison of the structures of these two classes of chorismate utilizing enzymes provides a rationale for the differences in quaternary structures seen for these enzymes, and indicates that the weak or transient association of  **PabB**  with  **PabA**  during catalysis stems at least partly from a limited interface for protein interactions.  Ref: 11841211 Biochemistry, 2002
- In the first step, a glutamine amidotransferase encoded by the  **pabA**  gene generates ammonia as a substrate that, along with chorismate, is used in the second step, catalyzed by aminodeoxychorismate synthase, the product of the  **pabB**  gene.  Ref: 11841211 Biochemistry, 2002
- **PabB**  glutamine enhances the protective effect of  **PabA** .  Ref: 7592344 J Bacteriol, 1995

**Non-interactive relationship** (e.g. studied together, co-existance, homology, etc.)

- Escherichia coli p aminobenzoate synthase is composed of two nonidentical subunits encoded by  **pabA**  and  **pabB**  and has been assumed to be the sole enzyme responsible for p aminobenzoate biosynthesis from chorismate and glutamine.  Ref: 2656685 J Biol Chem, 1989
- While there is much interest in the mechanism of chorismate aminations, there has been little work done on the ADC synthase  [ **PABB** ]   [ **PABA** ]  reaction.  Ref: 7592344 J Bacteriol, 1995
- These rules explain known trpG like fusions and explain why trpG and  **pabA**  are not fused to trpE and  **pabB** , respectively.  Ref: 2982857 J Biol Chem, 1985
- ... p aminobenzoate synthetase is composed of proteins encoded by  **pabA**  and  **pabB** .  Ref: 6330050 J Bacteriol, 1984
- Studies with the glutamine affinity label diazooxonorleucine DON reveal it is an inactivator of the glutaminase activity of the  **PabA**   **PabB**  complex, but DON does not alkylate and inactivate  **PabA**  alone.  Ref: 1637823 Biochemistry, 1992
- In contrast, unfused  **PabB**  group and fused  **PabA**  B proteins form a monophyletic group on a branch separate from the chorismate amination subunits of anthranilate synthases, probably reflecting a need for recognition of different positions in the common substrate chorismate.  Ref: 11981873 J Basic Microbiol, 2002
- Sub cloning the 4.5 kb fragment of S. griseus DNA into Escherichia coli  **pabA**  or  **pabB**  mutants by insertion at the BamHI site of pBR322 did not yield prototrophic clones directly.  Ref: 6420235 Gene, 1983
- The nt sequences indicated that  **pabA**  and  **pabB**  are closely linked in S. lividans and supported cloning evidence that the genes are expressed from a promoter with features resembling those of most E. coli promoters.  Ref: 8472953 Gene, 1993
- Aminodeoxychorismate synthase components I and II, encoded by  **pabB**  and  **pabA** , respectively, convert chorismate and glutamine to 4 amino 4 deoxychorismate ADC and glutamate, respectively.  Ref: 1644759 J Bacteriol, 1992
- Mutated  **PabB**  enzymes fall into three categories according to their properties deficiency of chorismate amination coupled with failure to associate with  **PabA** , deficiency of chorismate amination coupled with retention of  **PabA**  association, and competency of chorismate amination with failure of  **PabA**  association.  Ref: 8679677 Biochim Biophys Acta, 1996
- The Escherichia coli  **pabA**  and  **pabB**  genes have been overexpressed separately and in tandem.  Ref: 2251281 Proc Natl Acad Sci U S A, 1990
- In most bacteria, these two functions are encoded in separate genes e.g.,  **pabA**  and  **pabB**  of E. coli.  Ref: 11981873 J Basic Microbiol, 2002
- Equilibrium measurements were performed on the first partial ADC synthase  [ **PABB** ]   [ **PABA** ]  reaction at T=298.15 K and at pH=8.78.  Ref: 11975992 Biophys Chem, 2002
- We have determined that expression of a  **pabA**  lacZ chromosomal fusion is not changed by p aminobenzoate limitation, growth rate, catabolite repression, overexpression of either p aminobenzoate synthase subunit, or gene dosage of  **pabA**  and  **pabB** .  Ref: 2050628 J Bacteriol, 1991
- The nucleotide nt sequence of the 2.7 kb fragment contained two open reading frames, the deduced amino acid sequences of which were similar to those of  **pabA**  and  **pabB**  products from other bacteria.  Ref: 8472953 Gene, 1993
- This biosynthesis requires three separate proteins  **PabA** ,  **PabB** , and PabC.  Ref: 1637823 Biochemistry, 1992
- ... Pseudomonas TrpE and TrpG are more closely related to E. coli  **PabB**  and  **PabA**  than to E. coli TrpE and TrpG.  Ref: 2153661 J Bacteriol, 1990
- The protein encoded by ORF pab shows a clear relationship at the N terminus with  **PabA**  and at the C terminus with  **PabB**  from Escherichia coli, Serratia and Klebsiella.  Ref: 8472954 Gene, 1993
- The increased resistance was attributed to expression of the pair of discrete translationally coupled p aminobenzoic acid biosynthesis genes designated  **pabB**   **pabA**  cloned in the 3.8 kb fragment.  Ref: 11495989 Microbiology, 2001
- We report here the first successful copurification of  **PabA**  and  **PabB**  by gel filtration chromatography.  Ref: 8679677 Biochim Biophys Acta, 1996
- Comparison of the deduced amino acid sequence with database proteins indicated that the N and C terminal regions resembled  **PabA**  and  **PabB** , respectively, of numerous bacteria.  Ref: 8704974 Microbiology, 1996
- The overall biochemical reaction catalyzed by the  **PabB**  and PabC components of  **PABA**  synthase is chorismate aq ammonia aq =4 aminobenzoate aq pyruvate aq H 2 O l.  Ref: 11975992 Biophys Chem, 2002
- Genes involved in the biosynthesis of p aminobenzoic acid  **PABA**  in Streptomyces lividans 1326 were cloned in pBR322 by complementing a  **pabB**  mutant of Escherichia coli.  Ref: 8472953 Gene, 1993
- While various experiments have suggested that  **PabA**  and  **PabB**  act as a complex, attempts to isolate the intact complex have failed.  Ref: 8679677 Biochim Biophys Acta, 1996
- Introducing vectors containing the 3.8 kb NcoI fragment of S. venezuelae DNA into  **pabA**  and  **pabB**  mutants of Escherichia coli, or into the Streptomyces lividans pab mutant JG10, enhanced sulfanilamide resistance in the host strains.  Ref: 11495989 Microbiology, 2001
- H168Q has no catalytic activity and does not compete with wild type  **PabA**  for  **PabB**  this may indicate a folding defect.  Ref: 8096767 Biochemistry, 1993
- p Aminobenzoate synthesis in Escherichia coli requires three enzymes,  **PabA** ,  **PabB** , and PabC, acting respectively as glutaminase, chorismate aminase, and 4 amino 4 deoxychorismate aromatase.  Ref: 8096767 Biochemistry, 1993
- Together  **PabA**  and  **PabB**  convert glutamine and chorismate to glutamate and 4 amino 4 deoxychorismate.  Ref: 1637823 Biochemistry, 1992
- The 3840 bp DNA sequence fragment reported here contains a 2199 bp open reading frame encoding a 733 amino acid protein with similarity to the two components of  **PABA**  synthase described for prokaryotes Escherichia coli  **PabA**  and  **PabB** , suggesting that  **PABA**  synthase is bifunctional in yeast.  Ref: 8346682 Yeast, 1993
- Of these compounds, cycloheptadienyl analogue 11 has been found to be the most potent inhibitor of Serratia marcescens anthranilate synthase Ki = 30 microM for an RS mixture and of the E. coli  **pabB**  subunit of  **PABA**  synthase Ki = 226 microM.  Ref: 3311153 Biochemistry, 1987
- No inhibition of ADC synthase  [ **PABB** ]   [ **PABA** ]  activity was observed when p aminobenzoate, sulfanilamide, sulfathiazole, and several compounds requiring folate for their biosynthesis were used.  Ref: 7592344 J Bacteriol, 1995
- In this study,  **PabA**  from Escherichia coli has been purified to homogeneity from an overproducing construct and found to have no detectable glutaminase activity until addition of the E. coli  **PabB**  subunit.  Ref: 1637823 Biochemistry, 1992
- Using conditions established for maximal activity of the proteins, we measured the Km values for the glutamine dependent and ammonia dependent aminations of chorismate, catalyzed by  **PabB**  alone and by the ADC synthase  [ **PABB** ]   [ **PABA** ]  complex.  Ref: 7592344 J Bacteriol, 1995
- E170Q has no detectable glutaminase activity but does bind to  **PabB**  in competition with wild typoe  **PabA**  while E170K has neither detectable catalytic activity nor the ability to be recognized by  **PabB** .  Ref: 8096767 Biochemistry, 1993
- The association of  **PabA**  and  **PabB**  is greatly enhanced by the presence of 5 mM glutamine, and by preincubation at 37 degrees C.  Ref: 8679677 Biochim Biophys Acta, 1996
- Historically the gene products of  **pabA**  and  **pabB**  were assumed to be sufficient for de novo p aminobenzoate biosynthesis.  Ref: 2071583 J Biol Chem, 1991
- A 2.7 kb BamHI SstI fragment of the cloned DNA complemented  **pabA**  and  **pabB**  mutations in both E. coli and S. lividans.  Ref: 8472953 Gene, 1993
- The  **PabA**   **PabB**  complex has Kd less than 10 8 M but does not form a stable complex isolable by gel filtration.  Ref: 1637823 Biochemistry, 1992
